# Recent Advances in Multimodal Assessment Scoring Systems for Prostate Cancer: An Integrated Pathological and Imaging Perspective

**DOI:** 10.3390/diagnostics16142175

**Published:** 2026-07-13

**Authors:** Xiaoyun Zhang, Chaoqun Wang

**Affiliations:** 1 Department of Radiology, Affiliated Hainan Hospital of Hainan Medical University, Haikou 571199, China; 18898274202@163.com; 2 Department of Nuclear Medicine, Affiliated Hainan Hospital of Hainan Medical University, Haikou 571199, China

**Keywords:** prostate cancer, scoring systems, Gleason score, PI-RADS, PSMA-PET, multimodal scoring

## Abstract

In the clinical management of prostate cancer, continuously refined scoring systems form the cornerstone of precise risk stratification. Pathological assessment systems, represented by the Gleason score and the International Society of Urological Pathology (ISUP) grade groups, have long been the cornerstone of prostate cancer risk stratification; however, they are subject to inherent limitations such as sampling bias and spatial heterogeneity. With advances in imaging technology, the PI-RADS scoring system based on multiparametric magnetic resonance imaging (mpMRI) has been widely adopted for the detection and localization of prostate cancer; however, inter-observer variability remains a significant issue in clinical practice. Concurrently, emerging functional imaging scoring systems based on PSMA-PET, such as PSMA-RADS and miTNM staging, have provided new tools for the precise staging of prostate cancer and restaging following biochemical recurrence. Currently, an increasing number of studies are attempting to integrate pathological and multimodal imaging information to construct comprehensive predictive models, thereby addressing the shortcomings of single-modality assessment methods, achieving more personalized and precise risk stratification, and ultimately optimizing treatment decisions and improving patient prognosis.

## 1. Introduction

Prostate cancer is one of the most common malignant tumors in men, and its biological behavior is highly heterogeneous, ranging from indolent to highly aggressive disease [1]. This heterogeneity is evident not only in the clinical course of the disease but also at multiple levels, including genetic, molecular and metabolic [2,3]. For example, different anatomical regions of the prostate have varying rates of cancer development and different prognoses; tumors in the transitional zone generally respond better to treatment than those in the peripheral or central zones [1]. Even within the same Gleason grade 4 subtype—such as the expansive cribriform pattern—significant transcriptomic heterogeneity exists, suggesting that histological subtypes do not fully reflect biological diversity [4]. It is therefore essential to establish an accurate scoring system to distinguish between patients at different risk levels, guiding personalized treatment from active surveillance to curative therapy.

Traditional assessment relies primarily on PSA-based clinical staging, imaging-based anatomical staging, and pathological Gleason scores from prostate biopsy. However, these methods have limited capabilities when it comes to determining the spatial location of the tumor, assessing its volume, and identifying multifocal lesions. For example, the overall quantitative correlation between serum PSA levels and Gleason scores may be poor [5] and prostate biopsies are subject to sampling errors that result in grade changes in over 64% of patients after radical prostatectomy [6]. Furthermore, the Gleason system is inherently subjective, with inter-pathologist variability affecting its reproducibility [7]. These limitations have driven the development of more precise and comprehensive assessment tools. In recent years, advances in imaging technologies such as multiparametric magnetic resonance imaging (mpMRI) and prostate-specific membrane antigen positron emission tomography (PSMA-PET) have transformed the landscape of prostate cancer diagnosis and staging [8,9]. Multiparametric MRI (mpMRI), which combines T2-weighted imaging, diffusion-weighted imaging and dynamic contrast-enhanced imaging, has become a key tool in the initial diagnosis of prostate cancer, increasing the detection rate of clinically significant prostate cancer (csPCa) while reducing unnecessary biopsies and the overdiagnosis of clinically insignificant cancer [10,11]. To standardize mpMRI reporting, the Prostate Imaging Reporting and Data System (PI-RADS) was developed to assign a risk score—typically on a scale of 1 to 5—to lesions based on imaging characteristics [12]. The PI-RADS score correlates well with postoperative International Society of Urological Pathology (ISUP) grade groups and can predict positive surgical margins [13]. However, its positive predictive value (PPV) varies substantially across imaging centers, and its sensitivity and specificity for predicting features such as extracapsular extension remain suboptimal [12,14].

Concurrently, PSMA-PET/CT has emerged as a powerful molecular imaging tool that targets the PSMA protein on the surface of prostate cancer cells, demonstrating exceptional sensitivity and specificity in the detection of primary tumors, precise staging (particularly of lymph node and distant metastases), and localization of biochemical recurrence [15,16]. Compared with mpMRI, PSMA-PET offers significant advantages in detecting lymph node metastasis [17]. To standardize PSMA-PET interpretation and improve diagnostic accuracy for clinically significant cancer, the PRIMARY scoring system—based on lesion uptake patterns and intensity—was developed [18]. Studies have shown that combining PI-RADS with the PRIMARY score yields a composite (P) score with superior diagnostic performance, further improving clinical decision-making accuracy [19].

These standardized imaging-based scoring systems complement and validate classical histopathological systems, together forming the core of current multimodal assessment for prostate cancer. Building on this foundation, artificial intelligence (AI) and machine learning are being used to develop risk stratification models that integrate radiomics, pathological images, and even multi-omics data to address the challenges of tumor heterogeneity [20,21,22]. In addition, liquid biopsies—such as circulating tumor DNA and extracellular vesicles—and novel molecular biomarkers like lactylation-related genes provide new dimensions for risk stratification [23,24]. Recent advances in multimodal fusion have demonstrated substantial quantitative improvements over single-modality approaches: the P score achieved an AUC of 0.93 for csPCa detection, significantly outperforming PI-RADS alone (AUC 0.89) and PRIMARY alone (AUC 0.84), with a detection rate of 96–100% for high-grade lesions (P4-P5) [19]. Similarly, deep learning-based multimodal models integrating ^18^F-PSMA-1007 PET/CT and mpMRI have achieved internal and external validation AUCs of 0.877 and 0.872, respectively, for predicting high-risk biological features, surpassing clinical models (AUC 0.792) [22]. These quantitative metrics underscore the potential of multimodal integration to enhance risk stratification accuracy beyond traditional single-modality approaches. This article systematically reviews the evolution, clinical value, existing challenges, and integration trends of scoring systems across pathology, MRI, and PET, providing a comprehensive overview of research progress in this field.

### Methods

This review was conducted as a narrative review of the literature on prostate cancer scoring systems from a pathological-imaging integrated perspective. A literature search was performed in PubMed using combinations of the following keywords: “prostate cancer,” “scoring system,” “Gleason score,” “ISUP grade,” “PI-RADS,” “PSMA-PET,” “PRIMARY score,” “miTNM,” “multimodal integration,” and “artificial intelligence.” The search was limited to publications from January 2015 to June 2026, with no language restriction applied. Reference lists of retrieved articles were manually screened to identify additional relevant studies.

Studies reporting on the development, validation, or clinical application of pathological or imaging scoring systems for prostate cancer were included. Conference abstracts were not included due to insufficient data for critical appraisal.

## 2. The Evolution and Standardization of Pathological Scoring Systems

### 2.1. The Historical Evolution of the Gleason Scoring System and the 2014 ISUP Consensus

As the cornerstone of pathological grading for prostate cancer, the evolution of the Gleason scoring system reflects our deepening understanding of the biological behavior of tumors. The original Gleason scoring system is based on glandular patterns, with the total score (2–10) calculated by adding the primary and secondary patterns [25]. However, because the system relies on limited biopsy samples, its scores show significant discrepancies with postoperative pathology results. The study found that more than 64% of patients experienced postoperative Gleason grade upgrading compared with their preoperative biopsy results, highlighting the limitations of the original system in terms of precise risk stratification [6]. To this end, the International Society of Urological Pathology (ISUP) made significant revisions to the Gleason score at its consensus meetings in 2005 and 2014. In particular, the 2014 consensus eliminated low Gleason scores (2–5, e.g., 3 + 2 = 5) as an independent diagnostic category in clinical reporting and instead recommended the use of a prognostic grading system (Grade Groups 1–5); From a morphological standpoint, cribriform glandular structures are explicitly classified as a feature of high-grade carcinoma—all cribriform structures without comedo necrosis are classified as Gleason pattern 4, while those with comedo necrosis are classified as pattern 5 [26]. Studies have shown that tumors with an expansive cribriform (EC) structure are associated with poorer clinical outcomes following radical prostatectomy [4]. More importantly, the 2014 Consensus introduced the Grade Groups for prostate cancer, classifying Gleason scores of ≤6, 3 + 4 = 7, 4 + 3 = 7, 8, and 9–10 into Groups 1–5, respectively. This grouping system provides a more intuitive representation of the gradient of mortality risk; it has been adopted by the World Health Organization (WHO) and has been widely validated in clinical practice [26]. At the heart of this shift is a closer link between morphological patterns and specific clinical outcomes (such as prostate cancer-specific mortality).

### 2.2. Challenges and Scoring Systems in Pathological Assessment Following Neoadjuvant/Adjuvant Therapy

The pathological evaluation of prostate resection specimens following neoadjuvant endocrine therapy or radiation therapy presents unique challenges. Following treatment, tumor cells often undergo a series of treatment-related changes, such as cytoplasmic vacuolization and nuclear condensation; these changes make it extremely difficult to apply the traditional Gleason scoring system, which is based on glandular architecture. Distinguishing between the therapeutic effect and residual active tumors has become the central challenge in this scenario. For example, case reports indicate that even in patients who undergo robot-assisted radical prostatectomy and are found to have lymph node metastases in the preprostatic fat pad, postoperative pathological evaluation must carefully distinguish between the therapeutic effect and active tumor [27]. To this end, the ISUP recommends descriptive assessment using “therapeutic response” and evaluation of residual tumor morphology. However, no standardized quantitative scoring system has been established, representing a core challenge and research focus in current pathological assessment [28]. Although some studies have attempted to use semi-quantitative scoring based on the percentage of tumor cells or the worst residual pattern, their prognostic value still requires validation in prospective, large-scale cohorts. In particular, residual tumor burden (RCB) following neoadjuvant therapy has demonstrated potential for risk stratification—the 5-year metastasis-free survival rate was 100% in the RCB-0 group, compared with only 63% in the RCB-3 group. However, the clinical applicability of this classification system still requires further validation in independent cohorts [29]. Furthermore, studies have found that a combination of genetic variants in PTEN, AR-V7, TP53, TMPRSS2-ERG, and ERBB2 is significantly associated with tumor aggressiveness, suggesting that these variants may serve as potential biomarkers for risk stratification and personalized surgical decision-making in prostate cancer [30]. Therefore, the development of a comprehensive scoring system that integrates morphological descriptions of treatment effects, quantitative assessments of residual tumors, and potential molecular biomarkers is a key strategy for optimizing patient management following neoadjuvant or adjuvant therapy.

Although pathological scoring remains the reference standard for risk stratification, its inherent sampling bias and inability to capture whole-gland heterogeneity have prompted the development of imaging-based systems that can non-invasively assess the entire prostate.

## 3. Multiparametric MRI Scoring Systems: The Development and Clinical Application of PI-RADS

### 3.1. A Detailed Interpretation and Technical Highlights of PI-RADS v2.1

The Prostate Imaging Reporting and Data System (PI-RADS) is designed to standardize the acquisition, interpretation, and reporting of multiparametric magnetic resonance imaging (mpMRI); its core sequences include T2-weighted imaging (T2WI), diffusion-weighted imaging (DWI), and dynamic contrast-enhanced (DCE) imaging. Version 2.1, released in 2019, adjusted v2.0 to improve consistency and reproducibility in clinical practice [31]. The system defines primary diagnostic sequences based on anatomical regions: lesions in the peripheral zone (PZ) are primarily evaluated using DWI, while lesions in the transition zone (TZ) are primarily evaluated using T2WI. Each lesion is assigned a score of 1 to 5 based on imaging characteristics on the primary sequence (such as signal intensity, margins, and degree of diffusion restriction). A score of 3 indicates an “ambiguous” finding that requires careful clinical evaluation; a score of 4 indicates “possible clinically significant prostate cancer”; and a score of 5 indicates “highly likely clinically significant prostate cancer” [31,32]. By providing precise guidance for targeted needle biopsies, this scoring system not only improves the detection rate of clinically significant cancers but also effectively reduces the overdiagnosis of benign lesions, serving as critical evidence for clinical decision-making [10]. However, the effectiveness of the PI-RADS scoring system depends heavily on high-quality image acquisition; therefore, ensuring the quality of MRI images is of the utmost importance [33].

### 3.2. Clinical Validation, Limitations, and Future Prospects of the PI-RADS Scoring System

Numerous clinical studies have demonstrated that the PI-RADS score (particularly a score of ≥4) has high diagnostic accuracy for clinically significant prostate cancer (csPCa) [32]. For instance, a comprehensive 2024 systematic review and meta-analysis by Oerther et al., which synthesized data from 70 studies, confirmed this by reporting a pooled sensitivity of 89% and specificity of 66% for PI-RADS ≥ 4 at the patient level [34]. Multiparametric MRI can effectively identify aggressive tumors requiring intervention, thereby reducing unnecessary needle biopsies and significantly improving the detection rate of csPCa [35]. For example, in clinical practice, the PI-RADS score is often used in conjunction with prostate-specific antigen density (PSAD) to further optimize patient stratification and avoid unnecessary biopsies in low-risk patients [36]. However, the PI-RADS system also has certain limitations. First, there are interobserver differences; in particular, radiologists with varying levels of experience may reach different conclusions when interpreting “ambiguous” lesions, such as those in the transition zone (TZ) or those classified as PI-RADS 3. Second, the system exhibits significant variability in the positive predictive value for cancers with a Gleason score of 3 + 4 = 7, and it is challenging to assess lesions located in the anterior fibromuscular stroma [12]. Furthermore, although PI-RADS performs well in detecting localized lesions, its accuracy in predicting features of local progression, such as extra-prostatic extension (EPE), remains limited. Existing PI-RADS-based EPE prediction models have high specificity but low sensitivity [14]. Looking ahead, future developments for PI-RADS will include the integration of artificial intelligence (AI)-assisted scoring to improve the reproducibility and efficiency of interpretation [37]; explore quantitative thresholds for functional parameters (such as apparent diffusion coefficient [ADC] values) to provide a more objective basis for diagnosis [38]; and to assess the potential value of PI-RADS in active surveillance follow-up, such as using the PRECISE criteria to evaluate changes in serial MRI scans [39]. At the same time, by integrating PI-RADS with molecular imaging (such as PSMA PET/CT), we aim to develop a multimodal risk assessment model to achieve more precise risk stratification [19].

### 3.3. The Application of Other MRI Scoring Systems

As the clinical application of prostate MRI has expanded from diagnosis to comprehensive management, a series of scenario-specific scoring systems have been developed [39]. PI-QUAL is a 5-point scale used to assess MRI image quality and ensure diagnostic reliability. Studies have shown that lower PI-QUAL scores are associated with a higher proportion of PI-RADS 3 findings [40,41]. The PRECISE score is used for active monitoring of patients through serial MRI contrast-enhanced evaluations; a stable score (≤3) has a negative predictive value of 0.88 for disease progression. The PI-RR standardizes MRI assessment of local recurrence following curative surgery or radiotherapy, with an AUC ranging from 0.80 to 0.88 [40,42]. PI-FAB and PI-MAPS (under development) are designed for post-focal ablation and post-general ablation assessment, respectively [43]. These systems and PI-RADS together provide a complete MRI scoring framework for prostate cancer.

However, the information provided by mpMRI is largely confined to anatomical structure and functional parameters, and it does not capture the underlying molecular phenotype of tumors—a gap that PSMA-PET imaging, with its ability to target specific cell-surface biomarkers, has begun to address.

## 4. The Emergence and Application of PSMA-PET Imaging Scoring Systems

### 4.1. Standardized Reporting Systems for PSMA-PET: Whole-Body Assessment and Refined Intraprostatic Interpretation

With the widespread use of PSMA PET/CT in the staging of high-risk patients at initial diagnosis and in restaging following biochemical recurrence, standardized reporting systems have emerged to improve the consistency of image interpretation and the accuracy of clinical decision-making. In particular, the PSMA Reporting and Data System (PSMA-RADS) uses a 1–5-point scoring system to assess the likelihood of malignancy in lesions, providing clinicians with a clear grading of diagnostic confidence [44]. Different from the whole-body assessment of PSMA-RADS, the PRIMARY score was developed as a refined 5-level tool specifically for intraprostatic lesion interpretation, derived from the prospective multicenter PRIMARY trial. It classifies lesions by anatomical location and uptake intensity: score 1, no dominant pattern; score 2, diffuse transition zone (TZ) or symmetric central zone (CZ) activity not extending to the prostate margin; score 3, focal TZ activity (>2× background); score 4, focal peripheral zone (PZ) activity; and score 5, SUVmax > 12. In the original 291-patient cohort, csPCa detection rates increased progressively from 8.5% for score 1 to 100% for score 5 [18].

At the same time, the European Society of Nuclear Medicine (E-PSMA) and the Response Assessment in Neuro-Oncology—Prostate Cancer (RANO-PSMA) Working Group have proposed the “molecular imaging TNM” (miTNM) staging system, which aims to standardize the reporting of PSMA-PET findings and provide a unified framework for decision-making regarding metastasis-directed therapy and other treatment strategies [45]. These systems not only evaluate the primary prostate tumor but, more importantly, provide standardized descriptions of uptake and morphology in lymph nodes, bones, and visceral metastases, thereby enabling a precise assessment of tumor burden. For example, the PROMISE framework recommends quantifying tumor volume by organ system, reflecting the significant influence of metastasis site on biological aggressiveness [46]. Studies have shown that the interpretation of PSMA-PET images based on the miTNM and PSMA-RADS criteria exhibits high inter-observer agreement. The agreement was substantial for miTNM staging (Fleiss’ κ = 0.625–0.779) and almost perfect for PSMA-RADS scoring (intraclass correlation coefficient = 0.904), indicating that these systems offer good reproducibility [45]. The establishment of these standardized systems represents a crucial step forward in transforming PSMA-PET from an advanced diagnostic tool into a mature imaging biomarker that is integrated into multidisciplinary diagnostic and treatment workflows and guides precision therapy.

### 4.2. Clinical Value of PSMA-PET Scoring: From Biopsy Decision-Making to Treatment Response

Beyond its diagnostic utility, the PRIMARY score has also demonstrated value in informing biopsy decision-making. A Chinese dual-center study of 392 patients showed that the combination of PRIMARY score ≤ 3 and PSAD ≤ 0.2 ng/mL/cm^3^ achieved 100% specificity and positive predictive value for csPCa, sparing 54.1% of unnecessary biopsies in the discovery cohort (*n* = 243) and 69.6% in the external validation cohort (*n* = 149), with zero csPCa missed—substantially outperforming the EAU-recommended strategy of PI-RADS ≤ 2 + PSAD ≤ 0.2 (sensitivity 19.6%) [47]. This strategy is supported by the phase 3 PRIMARY2 trial, in which PSMA PET-CT avoided biopsy in 49% of participants (*p* < 0.0001) while maintaining non-inferior csPCa detection (12% vs. 16%; *p* = 0.0093), and reduced the detection of clinically insignificant cancer from 32% to 14% (*p* < 0.0001) [48]. In addition to its emerging role in biopsy decision-making, PSMA-PET has been extensively studied for disease monitoring after treatment, particularly in the setting of biochemical recurrence and therapeutic response assessment.

In patients with biochemical recurrence (BCR), the PSMA-PET score has become an important predictor of subsequent disease progression and survival. The visual analysis score, number, maximum standardized uptake value (SUVmax), and distribution pattern of the lesions are all closely associated with prognosis. A high tumor burden score, typically manifested as multiple metastases or a high SUVmax, is associated with a poorer prognosis [49]. For example, a retrospective study showed that among patients with biochemical recurrence, the positive detection rate of PSMA-PET increased with rising prostate-specific antigen (PSA) levels, reaching as high as 86% when PSA was ≥2 ng/mL [50]. In addition, quantitative parameters derived from PSMA-PET, such as PSMA tumor volume (PSMA-TV) and total-body PSMA burden (TL-PSMA), are being extensively studied for the assessment of early response and prognostic stratification in systemic therapies (such as novel endocrine therapies and ^177^Lu-PSMA radioligand therapy). These parameters provide a more objective way to quantify changes in systemic tumor burden. However, its clinical application still faces challenges: on the one hand, it is necessary to determine the optimal threshold for quantitative analysis; on the other hand, it is necessary to integrate PSMA-PET response criteria with those for conventional imaging (CT) and serum PSA. To this end, frameworks such as the PSMA-PET Progression (PPP) criteria and the PSMA Response Evaluation Criteria (RECIP) have been developed for different disease phenotypes, providing standardized tools for the objective assessment of treatment response [46]. These efforts aim to establish PSMA-PET as a powerful prognostic biomarker for dynamically monitoring treatment efficacy and guiding treatment adjustments.

## 5. Integration and Synergy of Pathology and Imaging Scoring Systems

### 5.1. Optimization of Pathological Scoring Through Image-Pathology Fusion-Guided Biopsy

MRI-US fusion-guided biopsy technology significantly improves the pathological assessment of prostate cancer by enabling precise sampling of suspicious areas (PI-RADS score ≥ 3) identified on multiparametric magnetic resonance imaging (mpMRI) [51]. This technique allows for the collection of tissue samples from specific suspicious lesions identified on imaging; however, studies have shown that standard systematic biopsy guided by transrectal ultrasound (TRUS) remains indispensable in patients with suspicious MRI findings, as it increases the detection rate of clinically significant prostate cancer (csPCa) [52]. Studies have shown that there may be discrepancies between the pathological results (such as Gleason scores) of targeted biopsies and systematic biopsies; clinical decisions should be based on the result indicating the highest risk. This demonstrates the value of imaging in spatially guiding pathological scoring, while also highlighting the complementary role of systematic biopsy to targeted biopsy. For example, in lesions with a PI-RADS score of 4, systematic biopsy may detect clinically significant cancer that was missed by targeted biopsy [53]. In addition, current research is exploring the development of a “combined imaging-pathology scoring” model based on quantitative parameters such as tumor-capsule contact length; the maximum length of contact between the tumor and the capsule has been shown to be an independent risk factor for predicting pathological upgrading (such as extracapsular invasion) and biochemical recurrence following radical surgery [54]. By integrating image-guided pathological information, it may be possible to more accurately predict the risk of pathological upgrade following radical prostatectomy, thereby providing a more reliable basis for personalized treatment decisions.

### 5.2. Development and Validation of an Integrated Clinical-Pathological-Imaging Model

Traditional clinical risk stratification models are being optimized using machine learning methods to improve risk prediction for prostate cancer patients [55]. For example, incorporating the PI-RADS score from prostate mpMRI or a positive result from prostate-specific membrane antigen positron emission tomography (PSMA-PET) into a predictive model can significantly improve the accuracy of predicting adverse outcomes such as pathological upgrading, extracapsular invasion, lymph node metastasis, and biochemical recurrence [56]. These integrated models use multivariate analysis to comprehensively evaluate traditional indicators such as PSA, clinical stage, and Gleason score, while also incorporating imaging features including lesion size, location, PI-RADS score, and the intensity of PSMA uptake (e.g., maximum standardized uptake value, SUVmax) [57,58].

In terms of specific implementation approaches, several research groups have explored different technical directions. Miao et al. [22] proposed a deep learning-based CL-MGNET model integrating ^18^F-PSMA-1007 PET/CT, mpMRI sequences, and clinical variables using a few-shot contrastive learning framework. Even with limited training data, the model accurately predicted high-risk biological features (ISUP ≥ 3, extracapsular extension, and positive surgical margins), achieving internal and external validation AUCs of 0.877 and 0.872, respectively, demonstrating robust cross-center generalizability. Telecan et al. [59] adopted a traditional machine learning approach, extracting radiomic texture features exclusively from T2WI sequences combined with clinical data (PSA, age, digital rectal examination) to construct a Random Forest classifier, achieving accuracies of 91.11% for distinguishing ISUP 1 from ISUP ≥ 2 lesions and 91.39% for distinguishing ISUP 2 from ISUP 3 lesions, demonstrating that even a single MRI sequence can achieve accurate preoperative grading. Liu et al. [60] further expanded the sample size to 1500 multi-center cases, employing deep learning to automatically segment the peripheral zone (PZ) and central gland (CG), extracting 12,918 radiomic features with multi-step selection to construct a prediction model achieving an AUC of 0.928 for six-class ISUP grading (0–5), effectively addressing the clinical implementation bottleneck of manual tumor delineation. These translational challenges are further discussed in Section 7.2.

## 6. Current Guideline Recommendations and Clinical Implementation

Contemporary guidelines increasingly recognize the value of integrating pathological and imaging scoring systems into routine prostate cancer management. The 2025 European Association of Urology (EAU) guidelines and the 2026 National Comprehensive Cancer Network (NCCN) guidelines provide evidence-based frameworks for the use of mpMRI, PI-RADS, PSMA-PET, and targeted biopsy [61,62].

### 6.1. EAU 2025 Guidelines

The EAU 2025 [61] guidelines strongly recommend performing pre-biopsy mpMRI in men with suspected organ-confined prostate cancer. For biopsy decision-making, when MRI is positive (i.e., PI-RADS ≥ 4), targeted biopsy should be combined with perilesional sampling. When MRI is negative (i.e., PI-RADS ≤ 2) and clinical suspicion of prostate cancer is low (PSA density < 0.20 ng/mL/cc, negative DRE findings, no family history), biopsy may be omitted in favor of PSA monitoring. For PI-RADS 3 lesions, biopsy decisions should be guided by PSA density: when PSA density is <0.10 ng/mL/cc and clinical suspicion is very low, biopsy may be deferred; higher PSA density thresholds (≥0.15–0.20 ng/mL/cc) indicate the need for biopsy.

For PSMA-PET/CT, the EAU recommends its use for metastatic screening in high-risk and locally advanced disease, and for restaging in biochemical recurrence when PSA > 0.2 ng/mL and results would influence treatment decisions. In patients with ISUP grade group 3 intermediate-risk disease, PSMA-PET/CT is recommended if available.

The updated EAU risk classification splits intermediate-risk disease into favorable and unfavorable categories. Active surveillance remains the standard for low-risk disease and may be offered to selected patients with favorable intermediate-risk features (ISUP grade group 2, <10% pattern 4, PSA < 10 ng/mL, low disease extent), whereas ISUP grade group 3 disease should be excluded from active surveillance protocols.

### 6.2. NCCN 2026 Guidelines

The NCCN 2026 [62] guidelines similarly prioritize mpMRI and PI-RADS in biopsy decision-making and risk stratification. PI-RADS ≥ 4 lesions warrant targeted biopsy, while PI-RADS 3 findings are managed through shared decision-making incorporating PSA density and patient preferences. Notably, the NCCN guidelines emphasize that PI-RADS 1–2 findings do not entirely exclude clinically significant prostate cancer, and systematic 12-core biopsy remains indicated when clinical suspicion persists.

For PSMA-PET/CT, the NCCN recommends its use for staging high-risk patients, restaging in biochemical recurrence, and selecting patients for ^177^Lu-PSMA-617 radioligand therapy in PSMA-positive metastatic castration-resistant prostate cancer. The NCCN risk classification stratifies patients into five categories (low, favorable intermediate, unfavorable intermediate, high, and very high risk), with active surveillance preferred for low-risk disease and selected favorable intermediate-risk patients with low-volume ISUP grade group 2 disease and low PSA density.

### 6.3. Commonalities, Discrepancies, and Current Gaps

**Figure 1:**
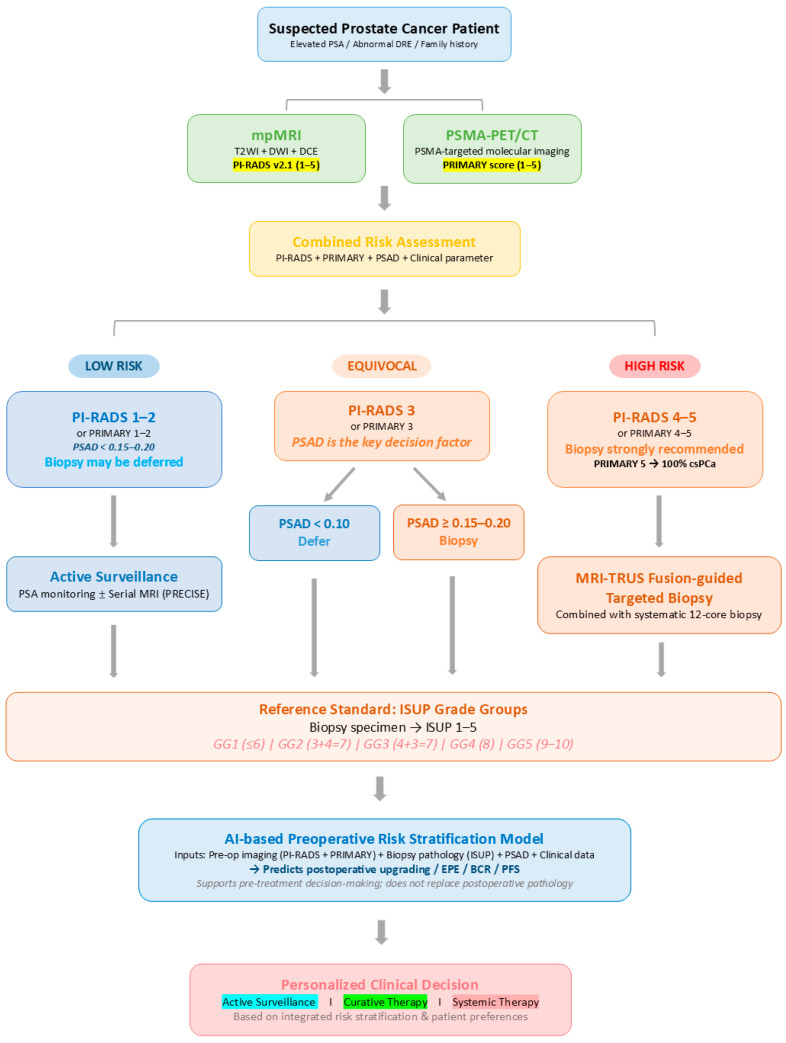
Multimodal diagnostic workflow and biopsy decision-making for prostate cancer.

Both guidelines concur on several core principles: pre-biopsy mpMRI is essential in the diagnostic pathway; PI-RADS provides a standardized framework for biopsy decisions; PSMA-PET/CT has transformed staging accuracy, particularly in high-risk and biochemically recurrent disease; and targeted biopsy improves detection of clinically significant prostate cancer while reducing overdiagnosis of indolent disease. Notable discrepancies include the EAU’s more detailed recommendations on integrating risk calculators and biomarkers into the diagnostic pathway, whereas the NCCN provides more granular treatment guidance for metastatic disease. Additionally, the NCCN has explicitly incorporated PSMA-PET for patient selection for radioligand therapy, a recommendation less prominently featured in the EAU document. Critically, neither guideline has yet incorporated composite or AI-based multimodal scoring systems—such as the P score (combining PI-RADS and PRIMARY scores) or deep learning-based integrated models—into formal recommendations. This gap reflects the need for large-scale prospective validation, standardized acquisition protocols, and demonstrated clinical utility and cost-effectiveness before these emerging tools can be integrated into routine practice. This translational challenge defines a key frontier for future guideline updates [61,62]. A proposed workflow integrating these scoring systems into clinical practice is illustrated in Figure 1.

The flowchart integrates PI-RADS and PRIMARY scoring systems with PSA density (PSAD) to guide biopsy decisions. Low-risk patients (PI-RADS 1–2 or PRIMARY 1–2, with low PSAD) may be considered for active surveillance and serial MRI follow-up. Equivocal PI-RADS 3 or PRIMARY 3 lesions require further stratification using PSAD: PSAD < 0.10 ng/mL/cc supports biopsy deferral, whereas PSAD ≥ 0.15–0.20 ng/mL/cc warrants biopsy. High-risk patients (PI-RADS 4–5 or PRIMARY 4–5) are recommended for MRI-TRUS fusion-guided targeted biopsy combined with systematic biopsy. Pathological ISUP grade groups serve as the reference standard. Following biopsy, AI-based models integrate preoperative imaging, biopsy pathology, and clinical data to predict postoperative pathological upgrading, extraprostatic extension, biochemical recurrence, and progression-free survival—supporting personalized treatment decisions before definitive therapy. The decision thresholds and workflow are derived from the EAU 2025 guidelines, NCCN 2026 guidelines, He et al. [47], and Buteau et al. [48].

## 7. Challenges, Controversies, and Future Directions

### 7.1. Heterogeneity Across Scoring Systems and the Standardization Dilemma

The core challenge currently facing multimodal assessment of prostate cancer lies in the significant heterogeneity among different scoring systems and the difficulties in standardizing their implementation. First, the use of pathological scoring systems, such as the Gleason score and the International Society of Urological Pathology (ISUP) grade groups, is complicated by discrepancies between preoperative biopsy findings and postoperative pathological results [6]. Imaging scoring systems, particularly the Prostate Imaging Reporting and Data System (PI-RADS), are also significantly influenced by the experience and expertise of the radiologist, and their positive predictive values vary widely across different medical centers, which undermines their reliability as standardized tools [12]. More importantly, there is often an imperfect correlation between histopathological findings and imaging phenotypes; for example, a lesion with a PI-RADS score of 5 may correspond to a Gleason score of only 3 + 4 on histopathological examination. This discrepancy is a common challenge in clinical decision-making [13]. At the same time, while emerging PSMA-PET scoring systems (such as PSMA-RADS and miTNM) have shown preliminary evidence of low interobserver variability, their clinical validation remains insufficient, and their performance across different cohorts requires further evaluation [45]. To overcome these subjective differences and achieve standardized and reproducible scoring, advancing the application of artificial intelligence in the digital analysis of histological slides and medical imaging has become a critical approach.

### 7.2. Artificial Intelligence in Prostate Cancer Scoring: From Technical Hurdles to Multi-Omics Integration

Although artificial intelligence has been proposed as a potential means of overcoming the subjective differences discussed above and achieving standardized scoring, the maturity and clinical translation of AI technologies themselves still face a series of practical challenges. First, heterogeneity in MRI acquisition protocols across institutions leads to systematic image differences that compromise the stability and comparability of radiomic features [60]. Second, segmentation variability—whether arising from inter-observer differences in manual annotation or from limited generalizability of automated segmentation algorithms—introduces uncertainty into feature extraction and compromises model performance [60]. Third, the reproducibility of radiomic features is a well-recognized challenge in the field: texture features extracted under different scanning conditions often exhibit poor reproducibility (intraclass correlation coefficient [ICC] < 0.5), directly challenging the cross-center generalizability of prediction models built on these features [63]. Finally, interpretability barriers remain a concern: the “black-box” nature of deep learning models may hinder clinical adoption, as physicians require a clear understanding of the model’s reasoning process to establish trust [22].

In addition, from a clinical translation perspective, several common bottlenecks remain. First, there are limitations in validation. Most models have undergone only single-center internal validation, lacking rigorous multi-center and prospective cohort testing; their generalizability across real-world multi-vendor equipment and heterogeneous scanning protocols remains unclear—among the three studies reviewed in Section 5.2, only Miao et al. [22] conducted preliminary external validation, albeit with only 36 external cases, limiting its conclusiveness. Second, information asymmetry between training labels and the prediction scenario may lead to systematic overestimation of model performance. All three studies used postoperative pathology as the gold standard training label, which is methodologically sound. However, the discordance rate between preoperative biopsy and postoperative pathology can be as high as 64% [6], indicating that postoperative pathology contains information inherently inaccessible to any preoperative assessment. Models may learn patterns associated with this “postoperatively available” information during training, which manifests as high AUCs in internal validation. Yet in the actual preoperative decision-making scenario, predictions must rely on the data actually available at the preoperative stage—primarily imaging and clinical parameters. This information asymmetry between the training and prediction scenarios suggests that model performance in real-world preoperative populations may be systematically lower than reported. Addressing these challenges will require multi-center standardized data acquisition, prospective clinical validation, and a paradigm shift in model evaluation—from technical performance toward clinical utility.

Despite these practical challenges, the future direction of the field remains clear. Future prostate cancer scoring systems designed for precision medicine will inevitably move beyond the current assessment frameworks, which focus primarily on morphology and macroscopic function, to integrate multidimensional molecular information from the genomics, transcriptomics, and proteomics fields. While the existing Gleason score and PI-RADS can reflect the morphological aggressiveness of tumors, they struggle to capture the underlying molecular biological mechanisms driving disease progression [4]. The future direction is to develop “digital biomarkers”—using artificial intelligence algorithms to extract deep features related to molecular subtypes, treatment response, and prognosis from pathological slides or imaging data such as MRI and PSMA-PET, thereby creating “imaging genomics” scores [20]. Research has shown that integrating multi-omics data (such as genomic variations and transcriptomic features) with clinical indicators through machine learning can more accurately predict postoperative progression-free survival in prostate cancer patients and enable more precise stratification of patients currently classified as high-risk [21]. In addition, liquid biopsy technology—particularly the analysis of exosomes, extracellular vesicles, and the nucleic acids, proteins, and other molecules they carry—offers the potential for non-invasive, real-time monitoring of tumor molecular characteristics [23]. The ultimate goal is to establish a dynamic, multidimensional “integrated scoring system.” This system will be capable of comprehensively integrating information on a patient’s histopathological features and genomic alterations (such as PTEN deletion, TP53 mutations, and AR-V7 expression) at various stages of diagnosis, treatment decision-making, and follow-up. By providing a comprehensive overview of a tumor’s biological behavior, it will offer a scientific basis for active monitoring, treatment selection, and recurrence prediction [1,30]. The key scoring systems discussed in this review are summarized in Table 1.

## 8. Conclusions

The evaluation system for prostate cancer has evolved from a model that relied solely on histological parameters to a dynamic, multimodal approach that integrates precise imaging, pathological grading, and molecular information. Classic pathological grading systems, such as the ISUP classification, have not lost their prognostic value; on the contrary, thanks to multi-parametric MRI-guided targeted biopsy, which allows for more precise tissue sampling and assessment, they remain the cornerstone of risk stratification. The PI-RADS scoring system provides a unified standard for the imaging detection and localization of clinically significant cancer, serving as a critical component of the diagnostic pathway. Meanwhile, PSMA-PET and its miTNM scoring system have led to substantial breakthroughs in disease staging—particularly in the precise detection and restaging of metastatic lesions—which directly influences the selection of treatment strategies.

The most notable change at present is that the two major fields of imaging and pathology are beginning to truly converge. The widespread adoption of MRI-ultrasound fusion-guided biopsy techniques, combined with comprehensive predictive models built on this foundation, has made personalized risk stratification possible. As a result, clinical decision-making now has a more reliable basis, allowing for more targeted approaches—whether opting for active surveillance, curative treatment, or systemic therapy.

The convergence of imaging and pathology has given rise to a practical tiered workflow. Given the current evidence, the clinical workflow follows a tiered logic: mpMRI and PSMA-PET (PI-RADS/PRIMARY) combined with PSAD for biopsy triage; ISUP grade groups as the definitive pathological reference standard; and AI-based models, while promising, remain investigational and require prospective validation before clinical adoption.

Of course, this multimodal evaluation framework still needs further refinement. First, scoring systems such as PI-RADS and various PSMA-PET interpretation criteria require further standardization and validation to reduce inter-reader variability and ensure the comparability of results across different hospitals. Second, integrating molecular biomarkers with clear prognostic value—such as genomic features and circulating tumor DNA—into existing scoring systems to create a truly practical “integrated score” remains a challenge that needs to be addressed. It is foreseeable that artificial intelligence will play a significant role in this field—through in-depth analysis of imaging, pathology, and multi-omics data, AI is expected to develop the next generation of dynamic, self-learning intelligent prognostic systems. These systems will not only improve predictive accuracy but may also reveal mechanisms underlying treatment response and drug resistance, ultimately driving the diagnosis and treatment of prostate cancer toward a more intelligent and forward-looking direction.

## Figures and Tables

**Table 1:** Multimodal Scoring Systems for Prostate Cancer.

Assessment Domain	Core Scoring System	Main Application	Correlation with Pathology/Imaging
**Pathology (Gold Standard)**	Gleason Score/ISUP Grade Groups	Diagnosis, prognosis	Cornerstone of grading: classifies scores ≤ 6, 3 + 4, 4 + 3, 8, 9–10 into GG1–5, reflecting mortality risk
	Gleason Pattern 4%/Cribriform	Tumor aggressiveness	Expansile cribriform pattern associated with worse outcomes; percentage of Pattern 4 affects prognosis
**MRI Scoring Systems**	PI-RADS v2.1	Diagnosis, risk stratification (1–5)	Predicts csPCa; score 3 equivocal, 4–5 highly suggestive
	PI-QUAL	Image quality control (5-point)	Lower score increases PI-RADS 3 findings, ensures diagnostic reliability
	PRECISE	Active surveillance (serial MRI)	Stable score (≤3) NPV 0.88 for disease progression
	PI-RR	Local recurrence after radical therapy	Diagnostic AUC 0.80–0.88, standardizes recurrence description
	PI-FAB/PI-MAPS	Post-focal ablation assessment	PI-FAB sensitivity ~93%; PI-MAPS under development
**PET/CT Scoring Systems**	PRIMARY Score	Intraprostatic PSMA-PET uptake (1–5)	Improves csPCa diagnosis, complements PI-RADS
	PSMA-RADS	Malignancy likelihood (1–5)	Provides diagnostic confidence, good reproducibility
	miTNM/PROMISE	Molecular staging, tumor burden	Standardized metastasis reporting; quantifies PSMA-TV, TL-PSMA by organ system
	RECIP/PPP	Treatment response (PSMA-PET)	Evaluates response to ^177^Lu-PSMA therapy, dynamic monitoring
**Multimodal Integration**	P Score (PI-RADS + PRIMARY)	Composite diagnostic score (5 levels)	Higher AUC (0.93 vs. 0.89/0.84), high-grade detection 96–100%
	MRI-US Fusion-guided Biopsy	Optimizes pathological sampling	Image-guided improves csPCa detection, reduces unnecessary biopsies
	Clinical-Pathological-Imaging Model	Predicts EPE, BCR, etc.	Integrates PSA, stage, Gleason, PI-RADS, SUVmax; machine learning enhances prediction

Note: csPCa, clinically significant prostate cancer; EPE, extraprostatic extension; BCR, biochemical recurrence; NPV, negative predictive value; AUC, area under the curve.

## Data Availability

No new data were created or analyzed in this study.

## Abbreviations

PSAD, prostate-specific antigen density; MRI, magnetic resonance imaging; TRUS, transrectal ultrasound; ISUP, International Society of Urological Pathology; AI, artificial intelligence; EPE, extraprostatic extension; BCR, biochemical recurrence; PFS, progression-free survival.
